# 

**Published:** 1919-10-18

**Authors:** 


### Matrons' and Sisters' Appointments.

Bradford City Hospital.—Miss A. Tinsdeall has been appointed night sister at the above hospital, where she received her fever training, and was afterwards staff nurse. She was trained at the Manchester Royal Infirmary, and subsequently became theatre charge nurse and holiday sister.

Leigh General Infirmary, near Manchester.—Miss E. Lloyd Rees has been appointed matron. She was trained at the Stanley Hospital, Liverpool, and has since been theatre and out-patient sister at the Accident Hospital, Ebbw Vale; sister and theatre sister at the West Cumberland Infirmary and at the Carnarvonshire and Anglesey Infirmary, Bangor; also matron at the latter institution.

Milford-on-Sea Cottage Hospital.—Miss H. E. Jobbett has been appointed sister-in-charge. She was trained at the Dreadnought Hospital, Greenwich, and has since held positions in the Endsleigh Palace Hospital, Euston, Hendon Cottage Hospital, and sister-in-charge at the Queen's Gate Hospital.

St. Mark's Hospital, City Road, E.C.—Miss A. R. Bunch, A.R.R.C., has been appointed assistant matron. She was trained at the General Hospital, Burton-on-Trent, and has since been staff nurse at the Brompton Hospital, London; staff nurse and acting sister at the General Hospital, Birmingham; sister T.F.N.S., 1st Southern General Hospital, Birmingham; housekeeping pupil and the charge of home sister's duties at Charing Cross Hospital.

Warneford, Leamington, and South Warwickshire General Hospital.—Miss T. Davey has been appointed matron. She was trained at the Royal Hospital, Sheffield, where she has been five years, and at the date of her new appointment held the position of acting matron.

### The College of Nursing (Limited by Guarantee).

### BIRMINGHAM CENTRE.

This Centre is actively engaged in collecting sufficient funds to start a club-room, where lectures can be held for the use of members and the exchange of social intercourse. An American Tea was arranged for September 18, which proved both enjoyable and successful, the balance in hand from the proceeds giving every encouragement for the success of future efforts for the same purpose. The Centre will hold its next meeting at the General Hospital on October 21, at 5 p.m. Mr. Bentson Hird, F.R.C.S., will lecture on the "Treatment and Nursing of Eye Cases." Non-members who are trained nurses will be welcomed. Admission 1s. each. Members free.

### EAST LANCASHIRE CENTRE (MANCHESTER BRANCH).

A very successful meeting of the above Centre was held at the Royal Infirmary, Manchester, on Tuesday, October 14, 1919, at 3.30 p.m., about seventy members of the College being present.

Mrs. Jessop Hulton, who had promised to address the members on "Special Features of the Present Divorce Bill," was unfortunately unable to attend, but very kindly arranged for Mr. Percy Glass, J.P., Chairman of the Lancashire County Higher Education Committee, to deliver a lecture on "The Evolution of Books." Mr. Glass's instructive and interesting lecture was greatly appreciated by the members present, and it is hoped that he will again address the members on this very interesting subject at some future date. Miss Mundy, in the absence of Miss Sparshott, kindly entertained the members to tea.

### BRIGHTON AND HOVE CENTRE.

A meeting will be held in the Residential Club at 45 Marine Parade on Friday, October 24, at 7.30 p.m. Miss Cowlin has kindly consented to give an address on "Nursing as a Profession." As this is to be the first meeting at the new club it is hoped that as many as possibly can will attend. Admission free to all nurses.

Appointment.—Miss A. M. Dawson has been appointed Lady Superintendent of the Residential Club for College nurses. Miss Dawson was trained at the Great Northern Hospital, where she was also housekeeping sister, and has been matron of Girton College.

### Passing Events and Topics.

### His Own Epitaph.

Dr. Robert Esler, of Queen's Road, Peckham, S.E., formerly a President of the Ulster Medical Society, and a surgeon of the Metropolitan Police, and author of a treatise on the Disposal of the Dead, has given directions in his will that he shall be buried in a plain perishable coffin, dressed in his evening suit, as in life, and that a plain marble stone shall be erected over his grave bearing his name and the following inscription: "Peace, peace. He is not Dead, he doth not Sleep; he hath awakened from the dream of life."

### Chingford's Memorial Hospital.

Chingford has decided that its war memorial shall take the form of a cottage hospital and the erection of a Celtic Cross. The scheme is well under way, and already some £1,700 has been received for the former object.

### Surgeon Flies to his Patient.

Not only as a means of postal transport, but also as a passenger "vehicle," has the aeroplane come to the fore during the railway strike, and not the least interesting incident in that connection is that of the case of a Harley Street specialist, who, it is stated, unable to obtain a train on account of the dislocation of traffic, flew from Hendon one day last week to perform an operation many miles away.

### Great Northern Central Hospital.

The Prince of Wales has consented to preside at a festival dinner in aid of the Great Northern Central Hospital early in December.

### Jubilee of National Children's Home.

The National Children's Home and Orphanage celebrates its jubilee this autumn. From a humble beginning in an obscure street off Waterloo Road the Rev. Dr. Stephenson founded the Home, which has now 3,300 children, and over 13,000 have passed through its hands. It has many interests and activities, having one branch on the South Coast for delicate children, an excellent sanatorium for incipient consumptives, another branch is devoted to the care of crippled children, while others are reserved entirely for war orphans and refugee children from Serbia. To commemorate the jubilee a sum of £150,000 is being raised to extend the accommodation of the Home.

### The Bishop of Kensington at St Mary's Hospital.

A new altar in the chapel of St. Mary's Hospital, Paddington, was dedicated by the Bishop of Kensington on Sunday, October 12. The altar is of fumed English oak, with a mensa of white marble. Its cost has been provided by patients and friends of the hospital.

Three beds have recently been endowed at the Seamen's Hospital, two by the Ladies' Emergency Committee of the Navy League, and the other by Mrs. Angus, the donor of the Convalescent Home. The new hospital for tropical diseases in Endsleigh Gardens will shortly be opened for the reception of patients.

### "Eclipse" Hot-water Bottle.

At this time of year the shops are flooded with india-rubber hot-water bottles, many of them remarkably tempting by reason of the low price at which they are offered. We recommend our readers, however, to pay careful attention to various points in making their selection, and to purchase bottles by reliable makers only, or disappointment will result. Ingram's "Eclipse" india-rubber hot-water bottles may be safely trusted on account of their well-constructed necks. Not only does their patent rubber-covered screw-stopper render leakage at this point impossible, but the patent constructed neck has a brass socket embedded in rubber, with the result that the weakest spot in the life of a rubber bottle is materially strengthened and the formation of the patent socket facilitates the filling process.

